# Four new species from the diatom (Bacillariophyceae) genus *Adlafia* Moser, Lange-Bertalot & Metzeltin from waterbodies of Vietnam

**DOI:** 10.3897/phytokeys.162.57657

**Published:** 2020-10-07

**Authors:** Anton M. Glushchenko, John Patrick Kociolek, Irina V. Kuznetsova, Maxim S. Kulikovskiy

**Affiliations:** 1 K. A. Timiryazev Institute of Plant Physiology RAS, IPP RAS, 35 Botanicheskaya St., Moscow, 127276, Russia; 2 Museum of Natural History, Boulder, Colorado, USA; 3 Department of Ecology and Evolutionary Biology, University of Colorado, Boulder, Colorado, 80309, USA

**Keywords:** *
Adlafia
*, diatoms, morphology, new species, Southeast Asia, Vietnam

## Abstract

Four species of the diatom genus *Adlafia* were found from waterbodies of Vietnam and described as new to science. Their formal descriptions are presented herein and they are illustrated by light and scanning electron micrographs. These new species are: *A. lamdongiensis* Glushch., Kulik. & Kociolek, **sp. nov.**, *A. babeiensis* Glushch., Kulik. & Kociolek, **sp. nov.**, *A. vietnamensis* Glushch., Kulik. & Kociolek, **sp. nov.** and *A. dauiensis* Glushch., Kulik. & Kociolek, **sp. nov.** These species are then compared to other similar taxa. Our new findings add to the number of species in this interesting genus and contribute to our understanding of the unique diatom flora found in Vietnam.

## Introduction

The genus *Adlafia* was proposed by Moser et al. (Moser et al. 1998). According to the original description, the genus is overwhelmingly represented by small-cell species (less than 25 µm in length). The raphe is naviculoid; external distal ends are smoothly bent and slightly extend to the mantle externally while the external proximal ones are drop-shaped, slightly bent to the opposite side from the distal ends (Kulikovskiy et al. 2016). On the inside, the raphe is located on a raised sternum, the distal ends with small helictoglossae, the proximal ends are straight and bent to one side (Morales and Le 2005). A distinctive feature of the genus is the presence of large, often square areolae, closed externally with a hymen and a continuous silica layer (Moser et al. 1998; Lange-Bertalot 2001). Species of the genus are distinguished from those in the genus *Kobayasiella* Lange-Bertalot in Lange-Bertalot and Genkal (1999) by lacking an “umbilicus”, a deflection or nick in the raphe system on the exterior. Currently, the genus belongs to taxa with an unclear taxonomic position (*incertae sedis*). Molecular studies of the genus require the involvement of more strains (Kulikovskiy et al. 2016).

The genus includes 27 species and infraspecific taxa (Guiry and Guiry 2020). Species of the genus are distributed around the world. Most species are aerophilous, being found mainly on mosses, but others prefer oligotrophic streams and lakes with a slightly higher or lower pH value, but are rare in large rivers (Spaulding and Edlund 2009; Kulikovskiy et al. 2016; Cantonati et al. 2017). Species are also known from fossil sediments (Lange-Bertalot and Metzeltin 1996; Benson and Kociolek 2012).

Southeast Asia is a floristically interesting region, from which many new genera and species of centric and pennate diatoms have been described recently (see Gusev and Kulikovskiy 2014; Glushchenko et al. 2016, 2017, 2018, 2019; Kapustin et al. 2017, 2019; Liu et al. 2018; Kulikovskiy et al. 2019, 2020; Rybak et al. 2019). Several *Adlafia* species have been recorded previously from Southeast Asia. In Indonesia, for example, *Adlafia bryophila* (J. Petersen) Lange-Bertalot in Moser et al. 1998 and *Adlafia minuscula* (Grunow) Lange-Bertalot in Lange-Bertalot and Genkal 1999 have been reported (Bramburger et al. 2004). *Adlafia sinensis* Liu & Williams in Liu et al. 2017 was described from south-central China. They also provide a comparison of many *Adlafia* species. In Vietnam, Adlafia minuscula var. muralis (Grunow) Lange-Bertalot in Lange-Bertalot and Genkal 1999 was reported from reservoirs, but without an image to document the determination (Duong et al. 2006).

The aim of our work was to identify the species diversity of the genus *Adlafia* in freshwater ecosystems of Vietnam.

## Materials and methods

A list of all samples examined in this study with their geographic positions is presented in Table 1. The samples were treated with 10% hydrochloric acid to remove carbonates and washed several times with deionized water for 12 h. The samples were subsequently boiled in concentrated hydrogen peroxide (≈37%) to dissolve organic matter. They were then washed four times with deionized water at 12 h intervals. After decanting and refilling with up to 100 ml deionized water, the suspension was spread onto coverslips and left to dry at room temperature. Permanent diatom preparations were mounted in Naphrax. Light microscopic (LM) observations were performed with a Zeiss Axio Scope A1 microscope equipped with an oil immersion objective (× 100, n.a. 1.4, differential interference contrast [DIC]) and Axiocam ERc 5s camera (Zeiss). Valve ultrastructure was examined by means of a JSM-6510LV scanning electron microscope (IBIW, Institute for Biology of Inland Waters RAS, Borok, Russia). For scanning electron microscopy (SEM), parts of the suspensions were fixed on aluminum stubs after air-drying. The stubs were sputter-coated with 50 nm Au in an Eiko IB 3 sputter coater. Samples and slides are deposited in the public collection of Maxim Kulikovskiy at the Herbarium of the Institute of Plant Physiology Russian Academy of Science, Moscow, Russia. The number of examined valves is indicated in each description of the species. The average value of the valve length, width and striae density, as well as standard deviation were calculated using Microsoft Excel 2020. Terminology of the valve follows Moser et al. 1998; Lange-Bertalot 2001; Morales and Le 2005; Kulikovskiy et al. 2016; Tusset et al. 2017 and Ciugulea et al. 2019.

**Table 1.** List of samples examined in this study. Geographic locality of samples and measured parameters indicated.

Sample/Slide	Locality	Habitat	Coordinates	Altitude, m	Temperature, °C	pH	Conductivity, µS cm^-1^	Coll. date
00269	Lâm Đồng Province, Da Tien Reservoir	benthos	11°58.816'N, 108°26.987'E	1503	21.5	6.4	81	21.06.2012
00321	Khánh Hòa Province, Hòn Bà Nature Reserve, Dầu River	wet moss	12°06.768'N, 108°59.888'E	275	24	6.7	92	28.05.2012
00325	Khánh Hòa Province, Suối Tiên River	benthos and periphyton	12°12.199'N, 109°01.694'E	68	26	6.9	101	02.07.2012
02168	Bắc Kạn Province, Ba Bể Lake	benthos	22°23.605'N, 105°36.856'E	163	26	8.5	174	19.04.2015
03593	Khánh Hòa Province, Khe River	periphyton	12°16.735'N, 108°54.677'E	34	26.8	6.9	84	08.07.2010
04633	Khánh Hòa Province, Hồ Câu Đôi Reservoir	periphyton	12°15.750'N, 109°04.012'E	9	29	6.8	110	14.09.2010

## Results

**Division: Bacillariophyta Haeckel**

**Class: Bacillariophyceae Haeckel**

**Naviculaceae *incertae sedis***

### Genus: *Adlafia* Moser, Lange-Bertalot & Metzeltin in Kulikovskiy et al. (2016)

#### Adlafia lamdongiensis

Taxon classificationPlantaeNaviculalesNaviculaceae

Glushch., Kulik. & Kociolek sp. nov.

5F436F59-DB7F-5A79-B740-04E025EB0FE6

Figs 1 , 2

##### Holotype.

**Figure 1.**
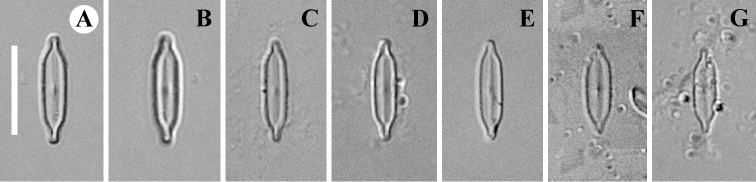
**A–G***Adlafia lamdongiensis* Glushch., Kulik. & Kociolek sp. nov. LM, DIC, size diminution series. Slides no 00269 (**A–C, E–G**) and 03593 (**D**). Holotype (**A**). Scale bar: 10 μm.

Slide no. 00269 in collection of Maxim Kulikovskiy at the Herbarium of the Institute of Plant Physiology Russian Academy of Science, Moscow, Russia, represented here by Fig. 1A.

##### Type locality.

Vietnam. Lâm Đồng Province, Da Tien Reservoir, benthos, 11°58.816'N, 108°26.987'E, 1503 m elev., *leg.* E.S. Gusev, *21.06.2012*.

##### Description.

**LM** (Fig. 1A–G). Valves linear with weakly convex margins. Ends are distinctly narrowly-rostrate. Length 9.7–13 μm (11.4 ± 0.9; n = 16), breadth 2.5–2.8 (2.7 ± 0.1; n = 16) μm. Striae and areolae not resolved in LM.

**Figure 2.**
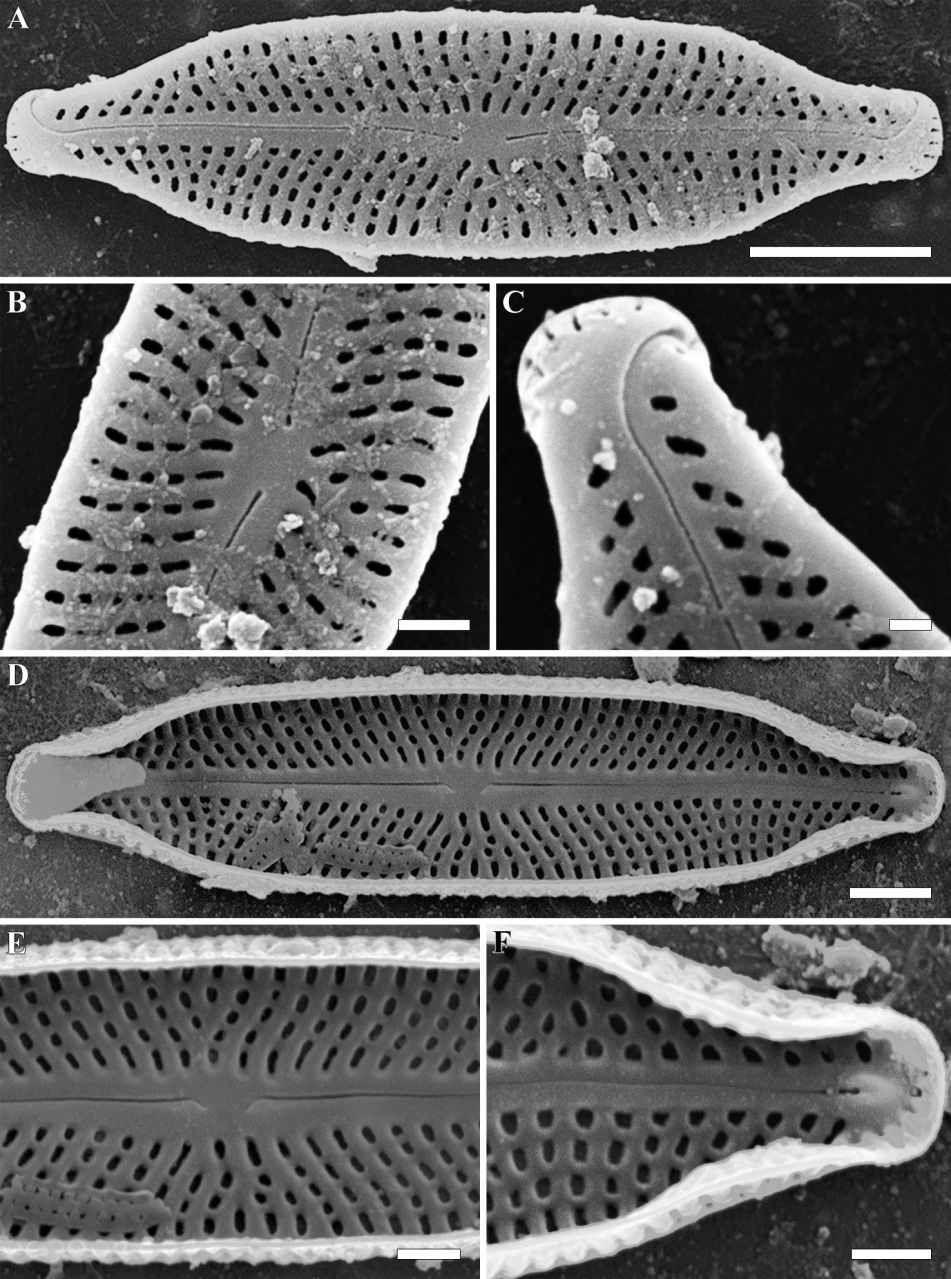
**A–F***Adlafia lamdongiensis* Glushch., Kulik. & Kociolek sp. nov. SEM. Sample no 00269 **A–C** external views **D–F** internal views **A** whole valve. The valve face is flat **B** central area **C** valve end **D** whole valve **E** central area **F** valve end. Scale bars: 2 μm (**A**), 1 μm (**D**), 0.5 μm (**B, C, E, F**).

**SEM, external view** (Fig. 2A–C). Valve face flat. Axial area linear. Central area absent. Raphe filiform, weakly lateral. Proximal raphe endings slightly expanded. Distal raphe endings positioned on valve mantle, hooked and curved in same direction, terminating at valve face-mantle junction. Striae uniseriate, radiate, becoming abruptly convergent approaching apices, Striae 45–50 in 10 μm (47.5 ± 1; n = 16). Areolae rounded or rectangular, hymenes not preserved during specimen preparation. Slit-like opening of apical areolae arranged in one row onto valve apex. Areolae 40–50 in 10 μm (45 ± 1.8; n = 16).

**SEM, internal view** (Fig. 2D–F). Raphe slightly lateral, lies in a prominent and raised raphe-sternum. Proximal raphe endings deflected towards primary side of valve. Distal raphe endings terminating in small helictoglossae. Striae continuing onto valve mantle. Short striae alternate with longer striae at valve center. Areolae rounded or rectangular. Openings of apical areolae apically elongated.

##### Etymology.

Epithet refers to the province of Vietnam (Lâm Đồng Province) where the specimens were found.

##### Distribution.

Vietnam. Type locality (slide no. 00269) and slide no. 03593.

#### Adlafia babeiensis

Taxon classificationPlantaeNaviculalesNaviculaceae

Glushch., Kulik. & Kociolek sp. nov.

6D928016-A673-5BF2-9F07-67EB3B1697E6

Figs 3 , 4

##### Holotype.

**Figure 3.**
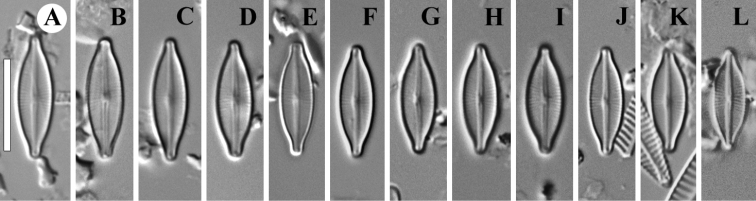
**A–L***Adlafia babeiensis* Glushch., Kulik. & Kociolek, sp. nov. LM, DIC, size diminution series. Slide no 02168. Holotype (**A**). Scale bar: 10 μm.

Slide no. 02168 in collection of Maxim Kulikovskiy at the Herbarium of the Institute of Plant Physiology Russian Academy of Science, Moscow, Russia, represented here by Fig. 3A.

##### Type locality.

Vietnam. Bắc Kạn Province, Ba Bể Lake, benthos, 22°23.605'N, 105°36.856'E, 163 m elev., *leg.* E.S. Gusev, *29.04.2015*.

##### Description.

**LM** (Fig. 3A–L). Valves lanceolate with rostrate ends. Length 11.5–14.0 μm (12.8 ± 0.6; n = 21), breadth 4.0–4.5 μm (4.3 ± 0.1; n = 21). Axial area narrow, almost linear. Central area weakly expressed or absent. Raphe filiform. Striae indistinct in LM, weakly radial at the central area, convergent towards to the ends. Areolae not resolved in LM.

**Figure 4.**
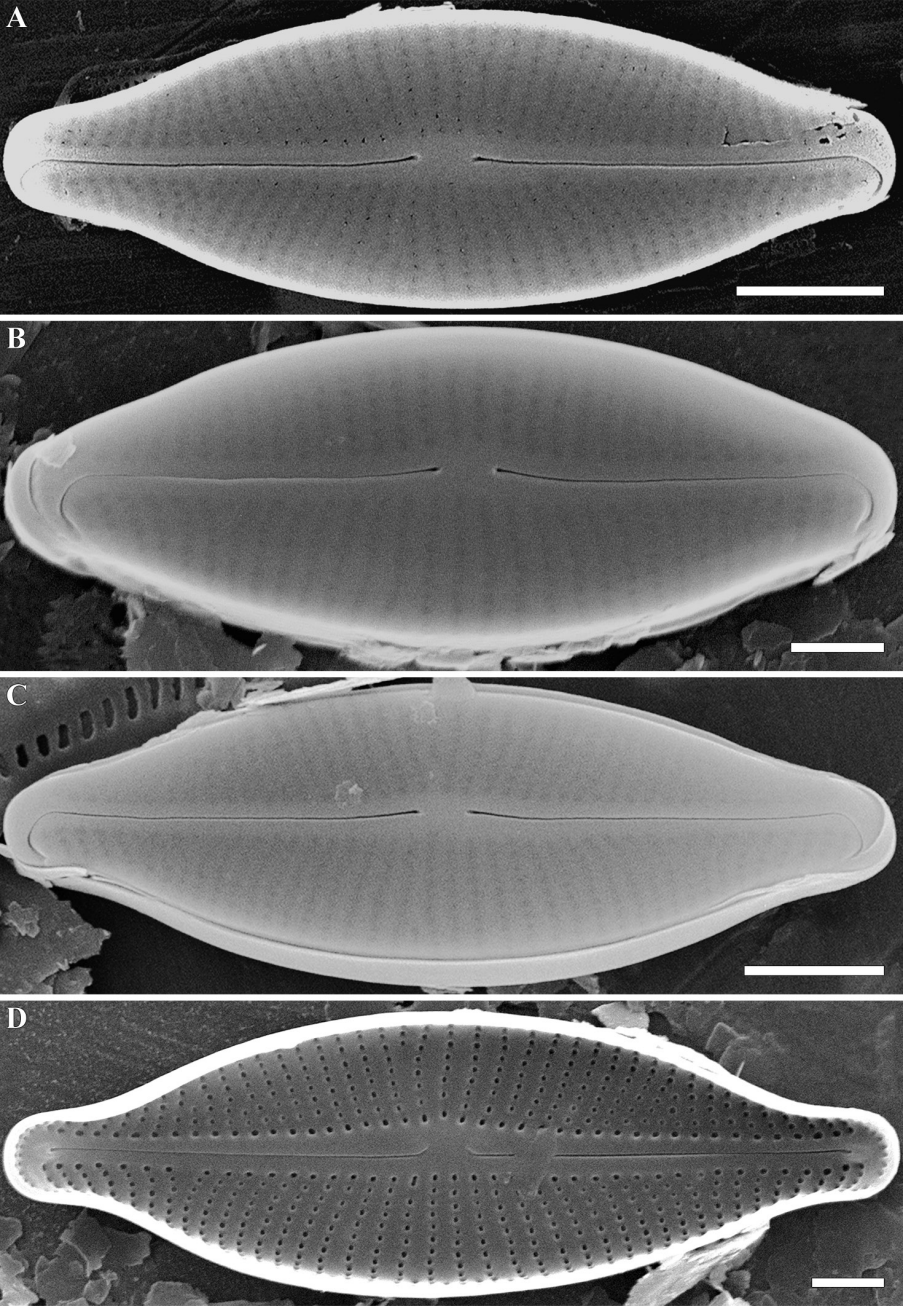
**A–D***Adlafia babeiensis* Glushch., Kulik. & Kociolek, sp. nov. **A–C**SEM, external views **D** internal views. Sample no 02168. Scale bars: 2 μm (**A, C**), 1 μm (**B, D**).

**SEM, external view** (Fig. 4A–C). Valve face flat. Axial area linear. Central area absent. Raphe filiform, weakly lateral. Proximal raphe endings slightly expanded, deflected. Distal raphe endings positioned on the valve mantle, hooked and curved in the same direction, and terminating at the junction valve face-mantle. Striae uniseriate, radiate, becoming abruptly convergent approaching apices, Striae 36–40 in 10 μm (38 ± 0.1; n = 21). Areolae rounded or rectangular, occluded by hymenes. Slit-like opening of apical areolas invisible. Areolae 65–70 in 10 μm (67.5 ± 0.8; n = 21).

**SEM, internal view** (Fig. 4D). The raphe straight, lying in a prominent and raised raphe-sternum. Proximal raphe endings deflected towards primary side of valve. Distal raphe endings terminating in small helictoglossae. Striae continuing on to valve mantle. Short striae alternate with longer striae at the center of the valve. Areolae rounded. Openings of apical areolae apically elongated.

##### Etymology.

Epithet refers to the lake of Vietnam where the new species was found.

##### Distribution.

Vietnam. Known only from the type locality.

#### Adlafia vietnamensis

Taxon classificationPlantaeNaviculalesNaviculaceae

Glushch., Kulik. & Kociolek sp. nov.

B3750889-8BF7-546E-8897-9AADA8807E56

Figs 5 , 6

##### Holotype.

**Figure 5.**
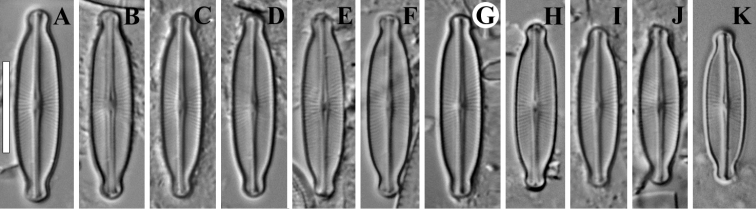
**A–K***Adlafia vietnamensis* Glushch., Kulik. & Kociolek, sp. nov. LM, DIC, size diminution series. Slides no 00325 (**B–K**) and 04633 (**A**). Holotype (**G**). Scale bar: 10 μm.

Slide no. 00325 in collection of Maxim Kulikovskiy at the Herbarium of the Institute of Plant Physiology Russian Academy of Science, Moscow, Russia, represented here by Fig. 5G.

##### Type locality.

Vietnam. Khánh Hòa Province, Suối Tiên River, benthos and periphyton, 12°12.199'N, 109°01.694'E, 68 m elev., *leg.* E.S. Gusev, *02.07.2012*.

##### Description.

**LM** (Fig. 5A–K). Valves linear-elliptical with capitate to subcapitate ends. Length 15–22 μm (18.5 ± 1.6; n = 20), breadth 3.5–5.0 μm (4.3 ± 0.4; n = 20). Axial area narrow, almost linear. Central area weakly expressed. Raphe filiform. Striae radiate, becoming abruptly convergent approaching apices, 32–34 in 10 μm (33 ± 0.4; n = 20). Areolae not resolved in LM.

**Figure 6.**
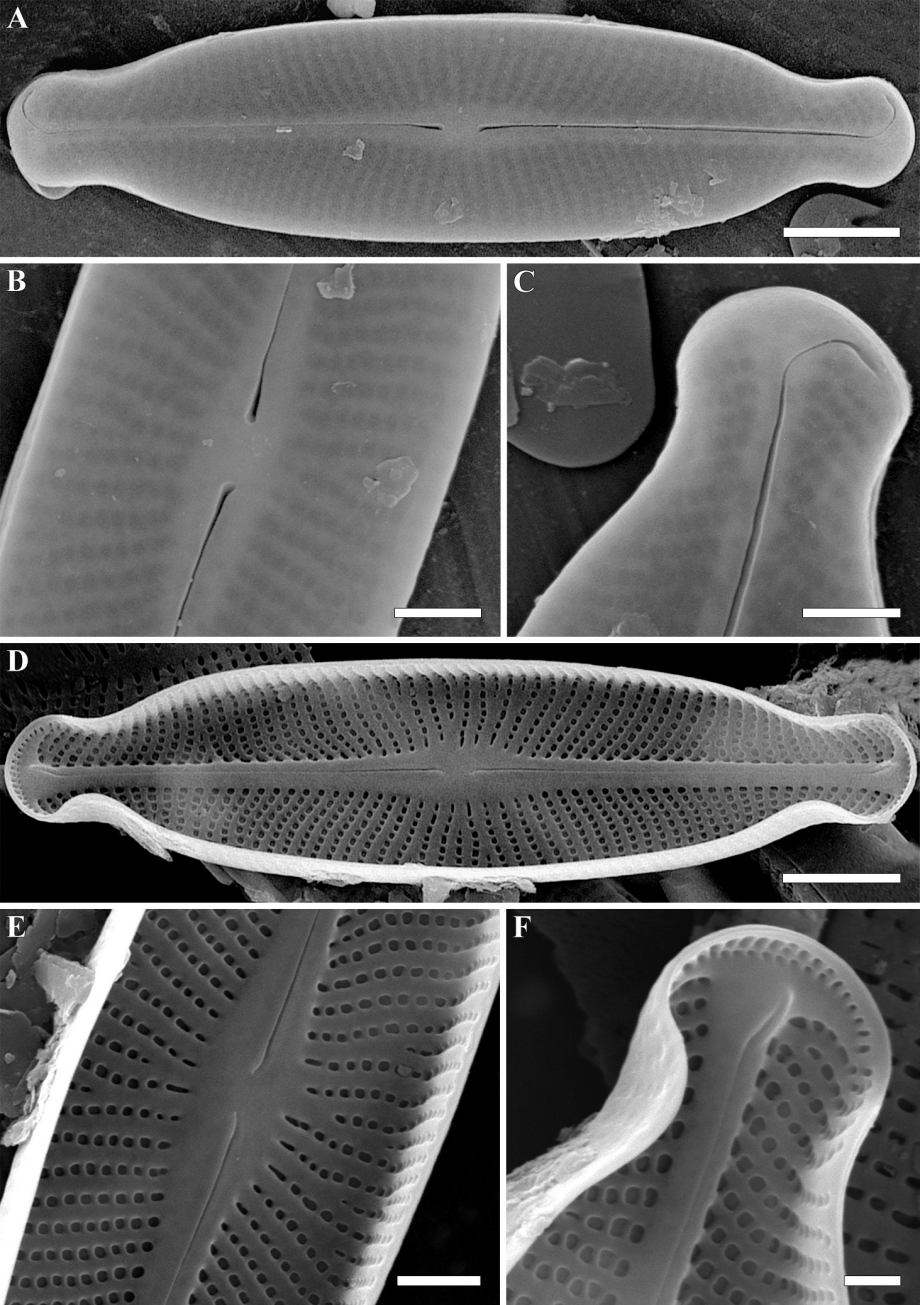
**A–F***Adlafia vietnamensis* Glushch., Kulik. & Kociolek, sp. nov. SEM, sample no 00325 **A–C** external views. **D, E** internal views **A** whole valve **B** central area **C** valve end **D** whole valve **E** central area **F** valve end. Scale bars: 2.5 μm (**A, D**), 1 μm (**B, C, E**), 0.5 μm (**F**).

**SEM, external view** (Fig. 6A–C). Valve face flat. Axial area linear. Central area weakly expressed. Raphe filiform. Proximal raphe endings slightly expanded, deflected. Distal raphe endings positioned on the valve mantle, hooked and curved in the same direction, and terminating at the junction valve face. Striae uniseriate. Areolae rounded or rectangular, occluded by hymenes. Slit-like opening of apical areolae invisible. Areolae 50–55 in 10 μm (52.5 ± 1.0; n = 20).

**SEM, internal view** (Fig. 6D–F). Raphe straight, lying in a prominent and raised raphe-sternum. Proximal raphe endings deflected towards primary side of valve. Distal raphe endings terminating in small helictoglossae. Striae continuing onto valve mantle. Short striae alternate with longer striae at the center of the valve. Areolae rounded or rectangular. The openings of apical areolae apically elongated.

##### Etymology.

Epithet refers to the country where the new species was found.

##### Distribution.

Vietnam. Slides no. 00325 (type locality) and no. 04633.

#### Adlafia dauiensis

Taxon classificationPlantaeNaviculalesNaviculaceae

Glushch., Kulik. & Kociolek sp. nov.

3F0E0B5B-55C7-5329-98B3-1E3187048AA7

Figs 7 , 8

##### Holotype.

**Figure 7.**
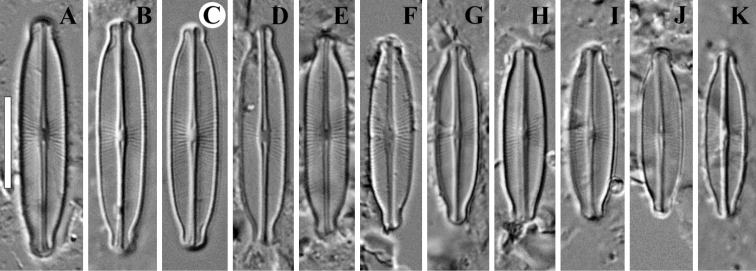
**A–K***Adlafia dauiensis* Glushch., Kulik. & Kociolek, sp. nov. LM, DIC, size diminution series. Slide no 00321. Holotype (**C**). Scale bar: 10 μm.

Slide no. 00321 in collection of Maxim Kulikovskiy at the Herbarium of the Institute of Plant Physiology Russian Academy of Science, Moscow, Russia, represented here by Fig. 7C.

##### Type locality.

Vietnam. Khánh Hòa Province, Hòn Bà Nature Reserve, Dầu River, wet moss, 12°06.768'N, 108°59.888'E, 275 m elev., *leg.* E.S. Gusev, *2.07.2012*.

##### Description.

**LM** (Fig. 7A–K). Valves linear to linear-elliptical with subcapitate ends. Length 19.0–26.5 μm (22.8 ± 2.3; n = 20), breadth 4.5–5.5 μm (5.0 ± 0.2; n = 20). Axial area narrow, almost linear. Central area weakly expressed. Raphe filiform. Striae radiate, becoming abruptly convergent approaching apices, 32–34 in 10 μm. Areolae not resolved in LM.

**Figure 8.**
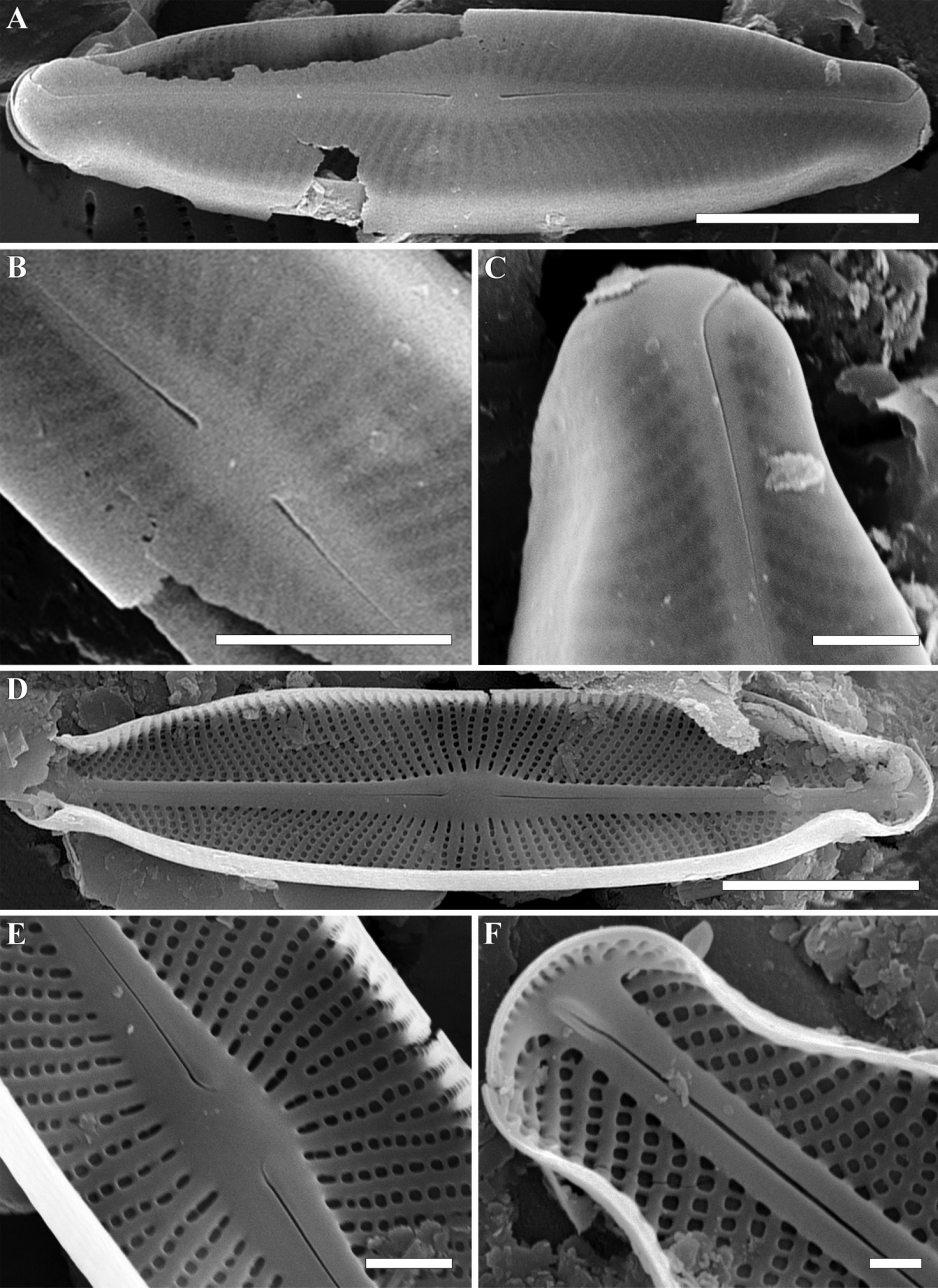
**A–F***Adlafia dauiensis* Glushch., Kulik. & Kociolek, sp. nov. SEM, sample no 00321. **A–C** external views **D, E** internal views **A** whole valve **B** central area **C** valve end **D** whole valve **E** central area **F** valve end. Scale bars: 5 μm (**A, D**), 2.5 μm (**B**), 1 μm (**C, F**), 0.5 μm (**F**).

**SEM, external view** (Fig. 8A–C). Valve face flat. Axial area linear. Central area weakly expressed. Raphe filiform. Proximal raphe endings slightly expanded, deflected. Distal raphe endings positioned on the valve mantle, hooked and curved in the same direction, and terminating at the junction valve face-mantle. Striae uniseriate. Areolae rounded or rectangular, occluded by hymenes. Slit-like opening of apical areolae invisible. Areolae 55–60 in 10 μm (57.5 ± 1.1; n = 20).

**SEM, internal view** (Fig. 8D–F). Raphe straight, lying in a prominent and raised raphe-sternum. Proximal raphe endings deflected towards primary side of valve. Distal raphe endings terminating in small helictoglossae. Striae continuing onto valve mantle. Short striae alternate with longer striae at the center of the valve. Areolae rounded or rectangular. The openings of apical areolae apically elongated.

##### Etymology.

Epithet refers to the river of Vietnam where the new species was found.

##### Distribution.

Vietnam. Known only from the type locality.

## Discussion

The four new species described here from Southeast Asia are morphologically similar to each other, but can be differentiated on the basis of valve shape, valve ends and striae density. All species share the morphological features typical for the genus *Adlafia*. A comparison of species to each other and with previously-described taxa shows that the new species from Southeast Asia are easily distinguished, unique taxa (Table 2).

*Adlafia lamdongiensis* sp. nov. resembles specimens identified by Lee as *Kobayasiella venezuelensis* Metzeltin & Lange-Bertalot (2007, p. 155, pl. 141, figs 10–23) specimens as illustrated with light micrographs (Lee 2012, fig. 15, K–M) on the basis of valve outline. Moreover, the valve identified by Lee in the SEM (Lee 2012, fig. 15, N) would appear to belong to the genus *Kobayasiella*, since there is a characteristic break of the raphe (the “umbilicus”) inherent to representatives of this genus. The valve has noticeably convex edges (Lee 2012, fig. 15, N), while in our material, and the light micrographs of Lee, valves are slightly convex. In our opinion, the light micrographs and a scanning image of Lee (2012) belong to species from different genera.

**Table 2.** Morphometric features of new *Adlafia* species and comparison with similar taxa.

Taxon	Outline	Valve ends	Valve length, μm	Valve width, μm	Striae in 10 μm	Areolae in 10 μm	References
*A. lamdongiensis* sp. nov.	linear with weakly convex margins	distinctly narrowly-rostrate	9.7–13.0	2.5–2.8	45–50	40–50	This study
*A. babeiensis* sp. nov.	lanceolate	rostrate	11.5–14.0	4.0–4.5	36–40	65–70	This study
*A. vietnamensis* sp. nov.	linear-elliptical	capitate to subcapitate	15–22	3.5–5.0	32–34	50–55	This study
*A. dauiensis* sp. nov.	linear to linear-elliptical	subcapitate	19.0–26.5	4.5–5.5	32–34	55–60	This study
*Adlafia multnomahii* Morales & Le	lanceolate	capitate to rostrate	9–16	4–5	37–45	65–70^*^	Morales and Le 2005
*A. detenta* Heudre, Wetzel & Ector in Ector et al.	elliptic to linear-elliptic	capitate	15–18	4.5–6.0	28–33	30–35	Heudre et al. 2018
*A. neoniana* Cantonati in Ciugulea et al.	elliptic-lanceolate	rostrate to subcapitate	9.4–18.5	3.7–5.1	30–32	45–50	Ciugulea et al. 2019
*A. decora* Tusset, Tremarin & Ludwig	linear-lanceolate	rostrate	18.2–26.2	4.6–5.7	24–32	50–54	Tusset et al. 2017
*Kobayasiella venezuelensis* Metzeltin & Lange-Bertalot *sensu* Lee	Linear with weakly convex margins^*^	subcapitate^*^	12.7–13.8^*^	2.7–2.8^*^	no data	no data	Lee 2012

^*^Data obtained from illustrations.

*Adlafia babeiensis* sp. nov. resembles *Adlafia multnomahii* Morales & Le (2005, p. 151, figs 1–38), differing from it mainly by having valves that are more lanceolate in shape and rostrate valve ends (Table 2). In *A. multnomahii*, on the other hand, the valve ends are capitate to rostrate. The density of striae in both species is similar (35–40 at 10 μm in *Adlafia babeiensis* sp. nov. in comparison with 37–45 at 10 μm in *A. multnomahii*). Our species also resembles *Adlafia detenta* (Hustedt) Heudre, Wetzel & Ector in Heudre et awl. (2018, p. 273), differing from it by the rostrate, rather than bluntly capitate, ends of the valves, striae that are more radiate in their orientation, higher density of striae (36–40 in our species versus 28–33 in 10 μm in *A. detenta*), and higher density of areolae (65–70 at 10 μm in our species versus 30–33 at 10 μm in *A. detenta*) (Table 2).

*Adlafia vietnamensis* sp. nov. resembles *Adlafia neoniana* Cantonati in Ciugulea et al. (2019, p. 381, figs 1, 2), by having more pronounced capitate ends, as well as less convex valves, in general, with a higher striae density (32–34 at 10 μm for our material compared to 30–32 at 10 μm for *Adlafia neoniana*) (Table 2). The density of the areolae of our species is also slightly higher (50–55 at 10 μm in *Adlafia vietnamensis* sp. nov. versus 45–50 at 10 μm in *A. neoniana*). *Adlafia vietnamensis* sp. nov. has a linearly elliptical shape of valves and valve ends from rostrate to subcapitate; *A. neoniana* is characterized by elliptical-lanceolate valves and rostrate to subcapitate ends. Our species is also similar to *Adlafia dauiensis* sp. nov. (see below) from which it differs mainly by a lower density of striae (50–55 in 10 μm in *A. vietnamensis* sp. nov. versus 55–60 in 10 μm in *A. dauiensis* sp. nov.). *A. vietnamensis* sp. nov. is slightly narrower than *A. dauiensis* sp. nov. (3.5–5.0 μm versus 4.5–5.5 μm). Valve ends of *A. vietnamensis* sp. nov. are capitate to subcapitate in shape while in *A. dauiensis* sp. nov. the ends are subcapitate. The outline of *Adlafia vietnamensis* sp. nov. is linear-elliptical, while *Adlafia dauiensis* sp. nov. has a linear to linear-elliptical outline (Table 2).

*Adlafia dauiensis* sp. nov. resembles *Adlafia decora* Tusset, Tremarin & Ludwig (2017, p. 261, figs 1–18), differing from it in having capitate, but not rostrate ends, as well as having less convex valves, with a slightly higher striae density (32–34 in 10 μm in our material in comparison with 24–32 to 10 μm in *A. decora*). The areola density is also different between the two species (50–54 at 10 μm in *A. decora* versus 55–60 at 10 μm in *A. dauiensis* sp. nov.) (Table 2).

These new species were found in different water ecosystems of Vietnam that show this genus is widespread in this country, especially in acidic ecosystems.

Morales and Le (2005) suggested *Adlafia* is a monophyletic group but they did not perform any formal analysis or present data to support their conclusion. Based only on a single species, Thomas et al. (2016) suggested *Adlafia* is part of a monophyletic group that could be considered the Cymbellales. No other analysis was forthcoming on this taxon, so this work did not address whether *Adlafia* is a monophyletic genus. Several authors, including in the original description of *Adlafia*, have made comparisons with *Kobayasiella* Lange-Bertalot in Lange-Bertalot and Genkal 1999 (as *Kobayasia* Lange-Bertalot, 1996, non *Kobayasia* S. Imai & A. Kawamura, 1958; see also Morales and Le 2005; Monnier et al. 2012; Van de Vijver et al. 2017). The two genera have fine striae, external distal raphe ends that are distinctly curved and external hymenate occlusions on the areolae. The difference between the two genera is usually suggested to be the absence (in *Adlafia*) or presence (in *Kobayasiella*) of a deflection (umbilicus) in the raphe system. However, this distinction has not always been applied consistently. For example, Le Cohu and Azémar (2010, figs 12, 13) showed specimens of *K. jaagi* (Meister) Lange-Bertalot, 1999 without the umbilicus. Liu et al. (2017) highlighted areas of the girdle that might help diagnose *Adlafia* as a monophyletic group, but these observations await formal analysis.

## Supplementary Material

XML Treatment for Adlafia lamdongiensis

XML Treatment for Adlafia babeiensis

XML Treatment for Adlafia vietnamensis

XML Treatment for Adlafia dauiensis
